# Family experiences of infant and young child feeding in lower-income countries: protocol for a systematic review of qualitative studies

**DOI:** 10.1186/s13643-016-0292-7

**Published:** 2016-07-08

**Authors:** Alessandra N. Bazzano, Aiko Kaji, Erica Felker-Kantor, Lisa Saldanha, John Mason

**Affiliations:** Department of Global Community Health and Behavioral Sciences, Tulane University School of Public Health and Tropical Medicine, New Orleans, USA

**Keywords:** Infant and young child feeding, Qualitative research, Nutrition, Low-income countries

## Abstract

**Background:**

Infant and young child feeding practices, including breastfeeding and complementary feeding of children under 2 years old, are crucially influenced by parent and family perceptions and experiences. Given the urgent need to improve nutrition of young children in low- and low-middle-income countries, both for reduction of morbidity and mortality in childhood and for future health outcomes, we propose to systematically review and synthesize available qualitative data specifically related to infant and young child feeding practices of parents and families in these settings, which may provide greater insights into barriers and facilitators to recommended feeding practices.

**Methods/design:**

The proposed study will systematically review existing qualitative research reporting infant and young child feeding practices from low- and low-middle-income settings. The Enhancing Transparency in Reporting the Synthesis of Qualitative Research (ENTREQ) statement will be used for reporting the stages of the review and dissemination. The search period will include all studies published from 2006 to 2016. The study selection process will follow established and recommended guidelines for reviews, and quality assessment will be conducted in two phases using critical appraisal and subsequently a confidence in findings approach derived from Grading of Recommendations, Assessment, Development and Evaluation-Confidence in the Evidence from Reviews of Qualitative (GRADE-CERQual). A full synthesis of the studies identified by the review will begin with thematic analysis and be followed by an interpretive approach to provide actionable information on the topic.

**Discussion:**

The findings will provide insight into the barriers and facilitators related to behavior that may hinder or enable implementation of interventions aimed at improving infant and young child feeding practices in lower-income settings.

**Systematic review registration:**

PROSPERO CRD42016035677

**Electronic supplementary material:**

The online version of this article (doi:10.1186/s13643-016-0292-7) contains supplementary material, which is available to authorized users.

## Background

The period from birth to 2 years of age is widely recognized as the most critical period during which nutrition may contribute to the survival, growth, and healthy development of children [1]. While maternal nutrition and exposures in utero are also vitally important to future health, an individual child’s nutrition during the first 2 years of life sets the stage for life course health development and may have direct implications for the health of future generations [2]. In addition to contributing to morbidity and mortality in childhood, it has been clearly established that early life nutritional exposures have lasting consequences and influence health trajectories and adult health outcomes like obesity, cancer, cardiovascular disease, and metabolic diseases [3, 4]. Economic development, demographic transitions, and global changes in dietary patterns mean that both undernutrition and overnutrition in early childhood present threats to future health.

The financial impact of improving young infant feeding practices cannot be understated. It has been estimated that not breastfeeding results in economic losses of $302 billion dollars annually [5], while stunting, one biomarker of chronic undernutrition in early life, continues to affect 159 million children [6]. While there have been international calls to action over the last 25 years to support improvement of early child nutrition [7] and particularly breastfeeding [8], which has decreased in low- and low-middle-income countries [9], progress continues to be inadequate [10].

The American Academy of Pediatrics [11], United States Surgeon General and Centers for Disease Control [12], and UNICEF and the World Health Organization [13, 14] all concur that optimal nutrition practices for childhood include exclusive breastfeeding for the first 6 months of life, followed by the addition of nutritionally adequate, safe, and appropriate complementary foods with continuation of breastfeeding for 1 year or longer. The United Nations Convention on the Rights of the Child states that every infant and child has a right to good nutrition [15]. Yet, in many low-income settings, these practices are not the norm, and despite international initiatives to improve infant feeding, an in-depth understanding of the complex biological and psychosocial underpinnings of infant and young child feeding remains out of reach.

Locally appropriate, high-quality counseling of mothers and other family and community gatekeepers, along with appropriate behavior change communication should be at the center of strategies to improve infant and young child nutrition as they are crucial for changing sub-optimal practices [16]. In order for those efforts to be successful, nutrition programs require behavioral and psychosocial information appropriate to context that can best be provided through qualitative research.

Thus far, no synthesis of existing qualitative data on family experiences around infant and young child feeding is available from lower-income settings to provide relevant information for the continued improvement of nutrition outcomes in low- and low-middle-income country settings.

### Objective

The primary objective of the proposed study is to systematically review qualitative literature related to family experiences of infant and young child feeding in low-income countries, synthesizing information on the barriers and facilitators that may impact interventions to improve nutrition and growth in childhood and, ultimately, health over the lifespan.

## Methods

This systematic review has been registered with the International Prospective Register of Systematic Reviews (PROSPERO): registration number CRD42016035677. The Preferred Reporting Items for Systemic Reviews and Meta-Analysis Protocols (PRISMA-P) checklist has been used in the preparation of this protocol and is included as an additional file (see Additional file 1).

### Study design

The review will follow the Enhancing Transparency in Reporting the Synthesis of Qualitative Research (ENTREQ) statement in reporting the stages of the review and dissemination. Due to the exclusive focus on qualitative research, the review will employ the ENTREQ guidelines [17] in lieu of the PRISMA guidelines, which are more specific to the requirements of quantitative literature reviews [18].

### Participants

Studies will be included which present data directly obtained from participants who were parents or family members of an infant or young child (0–2 years of age) at the time of the study. Family members may include mothers/fathers or other caregivers in the household who engage in infant or young child feeding. Infant and child feeding practices, defined for the purposes of the review, will include all actions taken to meet the physiological nutritional needs of children in this age group, such as the following: breastfeeding; introduction of solid, semi-solid, and/or family foods (known as complementary feeding); and continued breastfeeding of children in addition to providing solid/semi-solid food.

### Inclusion and exclusion criteria

Included studies will have used widely accepted qualitative data collection methods, with well-described methodology, including but not limited to the following: interviews, focus groups, direct observation, and participatory action research. Included studies will also have provided a clear description of recognized qualitative data analysis methods (e.g., grounded theory, narrative analysis, content analysis, thematic analysis).

Excluded studies will be those for which reviewers deem it will be difficult to extract qualitative data, e.g., mixed methods studies without clearly labeled data, or studies in settings where perceptions of parents/caregivers experiences of infant and young child feeding cannot be clearly identified, such as summaries or aggregate data. Commentaries will not be included. Additionally, studies from countries other than those defined by the World Bank as low-income countries and lower-middle income countries will be excluded [19].

### Intervention/exposure

For the purpose of this systematic review, infant and young child feeding practices will be defined as all actions taken by families that provide for the physiological nutritional needs of infants and young children 0–2 years old. These will include, but are not limited to, the infant and young child feeding practices defined by the international reference literature [20], breastfeeding for the first 6 months of life, introduction of complementary (solid/semi-solid) foods, and continued breastfeeding up to 2 years of age or beyond.

### Study setting

The study setting of interest to this review will be low- and low-middle-income countries. We will use the World Bank country and lending group definition of these, whereby countries are included which have a Gross National Income per capita of less than $4125 [19].

### Search strategy

The following electronic databases which are considered to be the most relevant for the topic will be searched: MEDLINE (PubMed); Embase; Cumulative Index to Nursing and Allied Health Literature (CINAHL: EBSCOhost); sociological abstracts through SocINDEX: EBSCOhost; and the Eldis knowledge-sharing web source for international development resources. The initial search strategy will be developed for MEDLINE and then adapted for the other databases. Medical Subject Headings (MeSH) will initially be used followed by free-text terms using controlled vocabulary (see the Appendix for a detailed description of the search strategy). Results will be restricted to English language publications from the last 10 years. The former limitation is due to potential difficulties in translating and interpreting foreign language qualitative data by native English-speaking reviewers, and the latter restriction is intended to ensure that the review identifies literature relating to the most current infant and young child feeding practices. In addition to the aforementioned search strategy, reference lists of included studies will be manually searched to identify any additional studies that fit the inclusion criteria.

In order to broaden the review’s applicability and not exclude important information from sources outside the peer-reviewed literature, we will include gray literature in the search strategy [21]. Given the large body of unpublished information relating to infant and young child feeding available through international agencies and non-governmental organizations, this will strengthen the relevance of the review.

For gray literature, we will initially draw up a list of relevant websites to search of organizations working in nutrition in lower-income countries (criteria for inclusion in this list will be developed in conjunction with experts working in the field). A custom search engine (CSE) will be created using Google Custom Search. Within the CSE, we will search the relevant websites identified in the first step using search strings adapted from those described above for databases. To refine the search strategy, we will screen the first 100 references (the first ten pages listed by relevance) obtained and proceed once a hit rate of 30 % has been achieved. We will also screen documents identified through a manual review of organizational reports and reports from relevant meetings. Results will be limited to publications in English from the last 10 years.

### Study selection

A flow diagram using PRISMA guidelines for reporting of systematic reviews will be used in reporting of the selection process and all results [22]. For organization of initial search results, Endnote reference management software (Thomson Reuters (Scientific) LLC) will be used, and results of searches imported to the software. At the first stage, duplicates and irrelevant studies will be removed. Two independent reviewers will then screen study titles and abstracts for suitability against pre-determined criteria for inclusion, and potential eligibility will be assessed. For the purposes of selection, three separate folders will be generated: one for studies that meet initial search criteria (where agreed by both reviewers), one for studies that do not meet criteria (where agreed by both reviewers), and one for further full-text review to determine eligibility. The decision to include or exclude a study in all cases must be agreed on by both reviewers. If after consultation a decision cannot be reached by the two reviewers, a third reviewer will make the final decision. For qualitative evidence synthesis, it may be necessary to obtain more papers in full text to assess eligibility against criteria, than would ordinarily be needed for a quantitative review.

## Quality appraisal

### Initial assessment

Each selected study will be initially assessed according to the Critical Appraisal Skills Program checklist [23] for quality and internal validity. In order to be considered of sufficient quality for review, selected studies will meet minimum criteria defined through the checklist. The stated criteria will include domains like appropriateness of study design, data collection techniques, and analysis methods used.

### Comprehensive assessment

At the second level of appraisal, we will use the GRADE-CERQual guidance [24] to identify which findings are strongly supported or less well supported. Two reviewers will independently review each study using guidance derived from GRADE-CERQual to reach consensus. In order to facilitate comparisons across the reviewed studies, a table presenting these findings will be created. This approach will allow for the findings to be appraised based on scores related to the confidence we can place in them, rather than excluding studies entirely. A table displaying the summary of findings using the CERQual approach will also be presented.

### Data extraction

For organization of extracted data, a unified matrix will be created and utilized to record specific characteristics of included studies. This will allow management and organization of the data to flow smoothly. Abstraction of data will be assigned to one reviewer, and a second reviewer will cross-check the abstracted data for accuracy. Extracted data will include, but not be limited to, reference details (author/data/publication), methodological approach (e.g., interviews/focus groups), conceptual basis underlying the study (e.g., Grounded Theory), objectives or aims of the study, sampling methodology, sociodemographic characteristics of participants, country/region, and analysis method(s).

## Analysis

We plan to conduct a further synthesis of the data following the review process. For the proposed synthesis, data from the results, discussion, and conclusion sections of the included studies, will be extracted into NVivo 11 qualitative software (NVivo qualitative data analysis software; QSR International Pty Ltd. Version 11, 2015) and thematic analysis employed to identify domains descriptive of the data for investigation and presentation. We will provide tables and visual representations of all data extracted and analyzed, including any data used for synthesis.

Thematic analysis will lead to a more interpretive phase in order to understand how the themes identified may represent barriers and facilitators to change in infant and young child feeding; for this we will use an approach similar to that of Thomas et al. [25]. In order to make the themes identified more actionable, reviewers will seek to infer barriers and facilitators during the synthesis process that are captured by the descriptive themes.

A narrative summary of themes identified through the initial analysis will be considered by three reviewers (independently at the first stage) to produce a listing of possible barriers and facilitators. At the next stage, reviewers will discuss and clarify the barriers and facilitators generated, so that more analytical themes may emerge. The theorized barriers and facilitators will then be considered alongside current interventions and approaches to improving infant and young child feeding (IYCF). Thus, an iterative process will be repeated until the themes to be presented in the synthesis are rendered sufficiently inclusive of all of the initial descriptive themes as well as the barriers and facilitators—and implications for intervention or program development—an approach that has been used elsewhere in synthesis [26].

## Discussion

While qualitative data on IYCF from lower-income settings is urgently required to improve the survival, health, and growth of current and future generations, a comprehensive synthesis of such information is currently lacking in the biomedical literature. Our proposed study will include a focus on the experiences and first-hand accounts of family members responsible for the nutritional needs of children under the age of 2 years. We anticipate that the findings will provide insight about the barriers and facilitators that hinder or enable positive changes in IYCF practices for better health.

### Strengths and limitations

Limitations of the study include the exclusion of documents not available in the English language and date limitations. Strengths of the proposed study include the use of multiple reviewers experienced in qualitative research and data collection and analysis, a comprehensive search strategy, assessment and scoring of quality and confidence placed in the findings based on guidelines, and a comprehensive description of all study findings.

## Appendix

### Sample search strategies

Sample strategy 1: “Infant Nutritional Physiological Phenomena” [Mesh] OR “Nutrition Physiology, Infant” [Mesh] OR “Infant Nutrition Physiology” [Mesh] OR “Physiology, Infant Nutrition” [Mesh] OR “Infant Nutritional Physiology”[Mesh] OR “Nutritional Physiology, Infant” [Mesh] OR “Physiology, Infant Nutritional” [Mesh] OR “Complementary Feeding” [Mesh] OR “Feeding, Complementary” [Mesh].

Sample strategy 2: “qualitative research” [MeSH Terms] OR qualitative[tiab] OR themes[tiab] AND ((“infant” [MeSH Terms] OR “infant” [All Fields]) AND (“Young Child” [Journal] OR (“young” [All Fields] AND “child” [All Fields]) OR “young child” [All Fields]) AND feeding [All Fields]).

Sample strategy 3 (free text terms to be used in varied combinations): nutrition, qualitative, formative, breast feed OR breastfeed, breast feeding OR breastfeeding, exclusive breast feeding, partial breast feeding, bottle feeding, breast milk substitute, complementary feeding, breast milk expression, weaning, infant and young child feeding, IYCF, infant OR child AND diet, infant OR child AND nutrition.

## Additional file

Additional file 1: PRISMA-P checklist. Completed checklist indicating inclusion of items noted. (DOCX 128 kb)
